# A phase I study of intratumoral ipilimumab and interleukin-2 in patients with advanced melanoma

**DOI:** 10.18632/oncotarget.10453

**Published:** 2016-07-06

**Authors:** Abhijit Ray, Matthew A. Williams, Stephanie M. Meek, Randy C. Bowen, Kenneth F. Grossmann, Robert H.I. Andtbacka, Tawnya L. Bowles, John R. Hyngstrom, Sancy A. Leachman, Douglas Grossman, Glen M. Bowen, Sheri L. Holmen, Matthew W. VanBrocklin, Gita Suneja, Hung T. Khong

**Affiliations:** ^1^ Division of Oncology, Huntsman Cancer Institute-University of Utah, Salt Lake City, UT, USA; ^2^ Department of Pathology, University of Utah, Salt Lake City, UT, USA; ^3^ School of Medicine, University of Utah, Salt Lake City, UT, USA; ^4^ Section of Surgical Oncology, Division of General Surgery Huntsman Cancer Institute-University of Utah, Salt Lake City, UT, USA; ^5^ Department of General Surgery, Intermountain Medical Center, Murray, UT, USA; ^6^ Department of Dermatology, Oregon Health & Science University-Knight Cancer Institute, Portland, OR, USA; ^7^ Department of Radiation Oncology, University of Utah, Salt Lake City, UT, USA

**Keywords:** ipilimumab, interleukin-2, intratumoral, abscopal effect, immunotherapy

## Abstract

**Purpose:**

Intratumoral interleukin-2 (IL-2) is effective but does not generate systemic immunity. Intravenous ipilimumab produces durable clinical response in a minority of patients, with potentially severe toxicities. Circulating anti-tumor T cells activated by ipilimumab may differ greatly from tumor-infiltrating lymphocytes activated by intratumoral ipilimumab in phenotypes and functionality. The objective of this study was to primarily assess the safety of intratumoral ipilimumab/IL-2 combination and to obtain data on clinical efficacy.

**Results:**

There was no dose limiting toxicity. While local response of injected lesions was observed in 67% patients (95% CI, 40%-93%), an abscopal response was seen in 89% (95% CI, 68%-100%). The overall response rate and clinical benefit rate by immune-related response criteria (irRC) was 40% (95% CI, 10%-70%) and 50% (95% CI, 19%-81%), respectively. Enhanced systemic immune response was observed in most patients and correlated with clinical responses.

**Experimental Design:**

Twelve patients with unresectable stages III/IV melanoma were enrolled. A standard 3+3 design was employed to assess highest tolerable intratumoral dose of ipilimumab and IL-2 based on toxicity during the first three weeks. Escalated doses of ipilimumab was injected into only one lesion weekly for eight weeks in cohorts of three patients. A fixed dose of IL-2 was injected three times a week into the same lesion for two weeks, followed by two times a week for six weeks.

**Conclusions:**

Intratumoral injection with the combination of ipilimumab/IL-2 is well tolerated and generates responses in both injected and non-injected lesions in the majority of patients.

## INTRODUCTION

With an estimated 73,870 new cases and 9,940 deaths, melanoma was the leading cause of skin cancer death in the US in 2015 [1]. For patients with localized melanoma the five year survival was 91% and for patients with regional and metastatic melanoma it declines to 63% and 16%, respectively [1].

Activation of transmembrane inhibitory receptor cytotoxic T lymphocyte associated antigen-4 (CTLA-4) *via* binding to B7.1 or B7.2 downregulates T-cell activation [2–6]. This results in inhibition of interleukin-2 (IL-2) secretion and T-cell proliferation. Additionally, CTLA-4 enhances the function of regulatory T cells (Treg). Blockade of CTLA-4 *via* anti- CTLA-4 antibody allows unopposed T-cell activation thereby breaking tolerance to tumor antigens [7, 8]. Ipilimumab (Ipi), a fully human IgG1 anti-CTLA-4 monoclonal antibody, was approved by the Food and Drug Administration (FDA) for metastatic melanoma in 2011 [9, 10]. Ipi lowers the threshold for T cell activation by blocking CTLA-4 expressed on activated T cells. Ipi has a response rate of approximately 11 % and is the first drug shown to significantly improve overall survival for metastatic melanoma [9, 11, 12]. However, since Ipi has limited tissue distribution and remains in the vasculature [13], circulating anti-tumor T cells activated by this drug may differ greatly from tumor-infiltrating lymphocytes (TIL) activated by intratumoral (IT) Ipi in terms of quantity and quality. With systemic Ipi associated with a low response rate and life threatening toxicities, [14] alternative combination and routes of administration of this drug are warranted.

Interleukin-2 (IL-2) is a glycoprotein discovered initially as a T cell growth factor [15, 16]. Activated CD4+ T cells, CD8+ T cells and dendritic cells (DC) are the main source of IL-2. IL-2 has been found to stimulate and enhance the function of cytotoxic T lymphocytes (CTL), natural killer cells and B cells [17–21]. While its in-vivo role is more complex, IL-2 plays key roles in driving T cell expansion, Treg function, and enhancing the differentiation, survival and effector function of long-lived memory CTL [22–25]. Administration of high dose systemic IL-2 was FDA approved for treatment of metastatic melanoma in 1998 [26]. The response rate of high dose IL-2 has been approximately 16%, with half achieving long term durable responses but it can cause severe toxicities [2]. To avoid toxicities, several studies have assessed the efficacy of IT IL-2 in melanoma patients [27–29]. While treatments were well tolerated, with only grades 1 and 2 toxicities and complete response of treated lesions in 62.5%- 69% of patients [27, 28], there were no systemic responses observed in uninjected lesions. The absence of abscopal effect, defined as a response in at least 1 non-injected lesion, may reflect a likely failure to boost systemic immunity by IT IL-2 despite an impressive local effect.

Intratumoral (IT) administration of Ipi has the potential to enhance local activation of tumor infiltrating lymphocyte (TIL) and effectively induce systemic activation of tumor-specific T cells. We hypothesized that the combination of IL-2 and Ipi administered IT would effectively hyper-activate TIL to induce a systemic immunity with minimal toxicities.

## RESULTS

### Patients

Twelve patients were treated at the Huntsman Cancer Institute, Salt Lake City, Utah between November 2012 and July 2014 in this phase I study. Nine of 12 patients had received prior treatment. The patient characteristics are listed in Table 1. The duration of exposure of the IT drug combination was 53 days. All patients receiving treatment were evaluated for dose limiting toxicities (DLT) during the first three weeks of treatment. The first three patients received IL-2 (3 mIU) and 0.5 mg Ipi over 8 weeks (dose level +1) as per protocol design. With 0 of 3 patients reaching DLT, three more patients were evaluated for dose level +2 of IL-2 (3 mIU) and 1 mg of Ipi over 8 weeks. With no DLT reported in any of the three patients in dose level +2, three more patients were evaluated at dose level +3 of IL-2 (3 mIU) and 2 mg of Ipi over 8 weeks. With no DLT reported and level +3 being the highest planned dose level, three additional patients were enrolled at the same dose level without evidence of DLT.

**Table 1:** Patient Characteristics (*n* = 12)

Characteristics	
**Sex**	
Male	6
Female	6
**Age (in years)**	
Median	63.5
Range	43-88
**Disease Stage**	
IIIb	1
IIIc	5
IV M1a	3
IV M1b	1
IV M1c	2
**Performance Status**	
ECOG 0	10
ECOG1	2
**Previous treatment**	
High dose interleukin-2	3
Systemic ipilimumab	4
Other therapy*	5

* Carboplatin, IT BCG, TIL therapy with fludarabine/cyclophosphamide pre-conditioning, GM-CSF, melphalan limb perfusion

### Toxicities

Of the 12 patients who received 216 individual treatments, none experienced DLT and all completed the treatment phase of the study. The patients were followed up on months 1, 4 and 7 after the last treatment. One patient in dose level +1 died during follow up, almost seven months since the end of his treatment, due to progression of disease and was not attributed to study medication. A total of 57 individual treatment-related AEs were reported. No patient in the study or the follow up phase developed a grade 4-5 AE. Treatments were well tolerated and toxicities are summarized in Table 2. The only related grade 3 toxicity observed was injection/tumor site ulceration/necrosis, which was not a DLT per protocol. One patient had grade 3 hyponatremia, which was present prior to treatment and thus unrelated. Other toxicities were grade 1 or grade 2 in nature. None of the toxicities could be clearly attributed to an autoimmune phenomenon.

**Table 2:** Treatment-related Toxicity (total events = 57)

Treatment related Toxicity	No. of instances of adverse event by grade (CTCAE V 4.0)			
**Toxic effect**	1	2	3	4
			
Anemia	1	0	0	0
Arthralgias	1	0	0	0
Cellulitis right leg	1	0	0	0
Chills	4	0	0	0
Creatinine increased	1	0	0	0
Crusting lesion (an injected lesion)	1	0	0	0
Dehydration	1	0	0	0
Dry mouth	1	0	0	0
Dry skin	1	0	0	0
Erythemia around scab	1	0	0	0
Eyelid Edema	1	0	0	0
Facial Swelling	1	0	0	0
Fatigue	6	0	0	0
Flu like symptoms	5	0	0	0
Hyponatremia	0	0	1	0
Injection site reaction	7	0	0	0
Itchy lesion	1	0	0	0
Nausea	1	0	0	0
Pain at injection site	5	1	0	0
Pruritis	1	0	0	0
Rash	2	0	0	0
Right eye edema- eyelid	1	0	0	0
Right femoral leg pain	1	0	0	0
Soft tissue infection	0	2	0	0
Swelling right ear canal	0	1	0	0
Tingling in right leg	1	0	0	0
Ulceration at injection site	0	0	5	0
White blood cell count decreased	1	0	0	0

### Tumor response

The secondary objective of this trial was to determine the clinical efficacy of the IT combination of IL-2 and Ipi. Ten of 12 patients were evaluable for objective response by immune-related response criteria (irRC). There were three partial response (irPR, 30%), one stable disease (irSD 10%), and six progression of disease (irPD, 60%). Hence, the overall objective response rate was 40% (95% CI, 10%-70%). This is summarized in Table 3. Two patients, who were unevaluable per irRC due to size criteria (lesions <10 mm), had regression of multiple skin lesions.

**Table 3:** Tumor Response [*n* = 12, 10 subjects evaluable, 2 subjects non-evaluable per immune related response criteria (irRC)]

Best response	Injected lesion	irRC	irRC with pathology correlation
CR	7/12	0/10	1/10
PR	1/12	3/10	3/10
SD	-	1/10	1/10
PD	4/12	6/10	5/10

Local response of the injected lesion, by measurement and/or pathology (resection or biopsy), was seen in eight patients (seven local complete response (CR) and one local PR, 67% (8/12) (95% CI, 40%-94%)). Four local PD was observed; however, these four lesions were not biopsied for confirmation.

Interestingly, an abscopal effect, was seen in eight of nine patients (88.9%) (95% CI, 68%-100%) at locoregional and distant sites. Three patients were not evaluable for abscopal effect since they had only one lesion at baseline.

Four of ten evaluable patients, patients 5, 8, 9 and 11, (40%) (95% CI, 10%-70%) achieved objective responses based on imaging and/or pathology. Patient 5, with progression on prior intratumoral BCG injections, was enrolled at dose level+2. She had a partial response at one month follow up, but chose to enroll in another trial even without disease progression. Therefore, duration of response was undeterminable in her case. She died from disease progression more than 19 months from her enrollment in our study.

**Figure 1:**
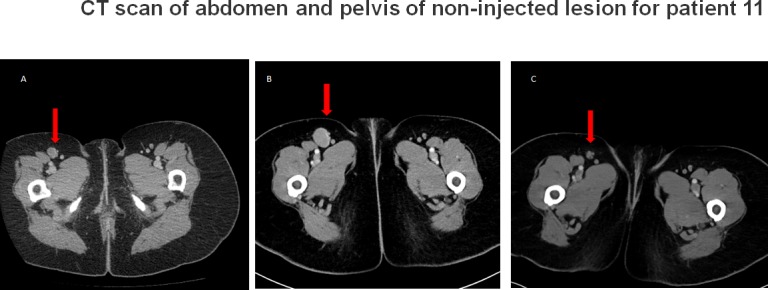
CT scan of abdomen and pelvis of non-injected lesion for patient 11 (shown by the arrow) at **A.** screening **B.** 1 months post last injection and **C.** 4 months post last injection. Initial progression followed by regression was observed in (B) and (C), respectively. This figure reflects both pseudo-progression and abscopal response.

Patients 8, 9 and 11 were enrolled in dose level +3. Patient 8 was found to have disease progression of the right supraclavicular mass and a new lesion based on physical examination and imaging at one month following the last injection. However, pathology of the resected lesions showed mixed inflammation and necrosis but no residual viable melanoma. Therefore, patient 8 had a pathologic CR despite being classified as irPD and continues to have no evidence of disease (NED). Patient 9, who had progressed on HD IL-2 prior to enrollment, achieved irPR. She had a residual melanoma lesion resected and has been NED since. Patient 11 had irPR as best response. The non-injected right femoral lymph node had increased by 147% of baseline at month 1 follow-up, without further treatment decreased by 75% of baseline at month 4 follow-up (Figure 1). This lymph node completely regressed with further follow up. We used a PR designation for her response since the CR occurred after the study follow up period. She continues to be free of disease. Thus 3/4 patients (75%) who had objective response by imaging and/or pathology are still alive and currently free of disease, more than 31+ months from their enrollment in our study. Of interest, these three patients were in the last cohort of six patients (50%) who received drug at the highest planned dose level.

### Immunologic response

In order to assess the systemic immune response, we examined the intracellular expression of IFNγ, granzyme B, perforin, Tbet and FoxP3 in circulating T cells in ten patients with adequate blood samples. An increase in frequency of CD8+ T cells expressing IFNγ and Tbet was observed in 6 patients (60%), with fold-increase of 2.02 (95% CI, 1.31-2.73) and 1.75 (95% CI, 1.14-2.36). An increase in frequency of CD8+ T cells expressing granzyme-B and/or perforin was observed in four (40%) and five (50%) patients, respectively, with fold-increase of 1.83 (95% CI, 1.61-2.05) and 1.50 (95% CI, 1.32-1.68), respectively.

**Figure 2:**
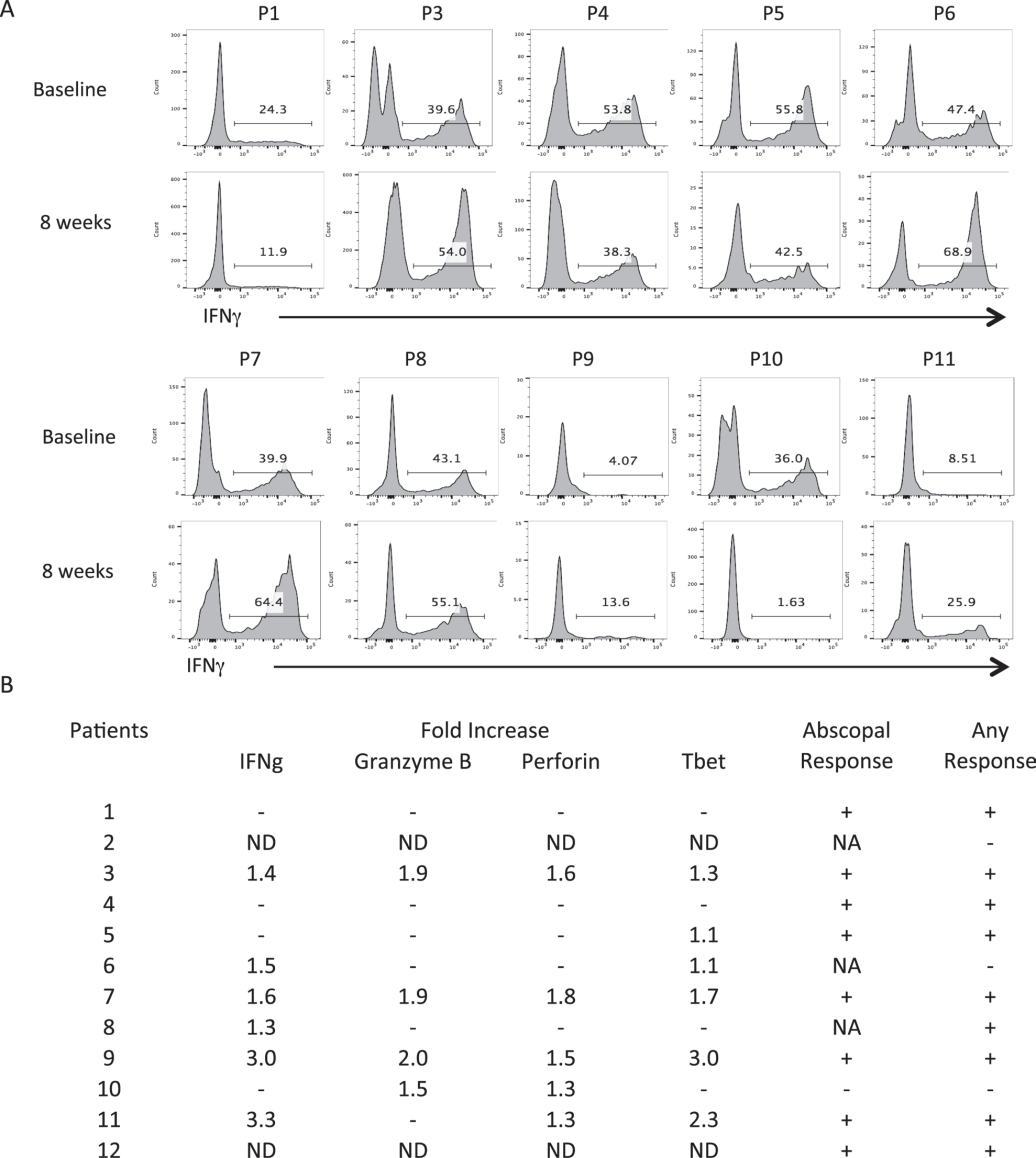
Circulating IFNg-producing CD8 T cells (P/I stimulation 4 h) PBMCs at baseline and end of treatment were analyzed by flow cytometry as described. Intracellular cytokine staining for factors relating to T lymphocyte activity were compared between the start and cessation of treatment for CD8+ CTLs **A.** Fold increase in the percentage of CD8+ cells that also stain positive for these markers over time are summarized in **B.** Fold increase in total circulating CD8+ T cells expressing IFNg: 2.02 (95% CI, 1.31-2.73); Granzyme B: 1.83 (95% CI, 1.61-2.05); Perforin: 1.50 (95% CI, 1.32-1.68); Tbet: 1.75 (95% CI, 1.14-2.36). ND: not done; NA: not applicable as the patient had 1 lesion at baseline. Any response includes objective, abscopal and/or pathologic response.

Seven patients (70%) had an increase in CD8+ T cells expressing at least one of these effector molecules. A similar pattern was also observed when correlated with patients who experienced any response. That is, six of nine (67%) patients with any response demonstrated increased levels of peripheral CD8+ T cells expressing at least one of these activation markers (Figure 2). There were no significant changes in CD4+ T cell parameters Foxp3 or IFNγ.

## DISCUSSION

This study was to determine the toxicities and clinical response associated with the IT injection of IL-2 and Ipi. Ipi while enhances the immune response against tumors [30] frequently unleashes immune related toxicities. Two of four patients in our study who had prior-systemic Ipi treatment had discontinued because of immune related toxicities. IT injection with the combination of Ipi/IL-2 was well tolerated, including those who already had prior exposure to systemic Ipi and high dose IL-2. Most toxicities were grade 1 or 2 in nature.

While clinical cases of abscopal effects in response to radiotherapy have been reported since 1973, more recently radiotherapy has been reported to be synergistic with immune stimulatory drugs like anti-CTLA-4 and anti-PD1 [31]. The inflammatory microenvironment and the cytotoxic immune system have been suggested as the main drivers of spontaneous regression [32–34]. In the present study an abscopal effect was seen in 8 of 9 patients (89%). Abscopal effect impacts distant tumor growth and originates, at least in part, from enhanced immune functions such as DC and CTL activation. DCs present tumor peptides *via* and deliver co-stimulatory signals to naïve CD4 and CD8 T cells that mediate tumor-specific cell killing [35].

We hypothesize that with IT Ipi/IL-2, T cells are activated at two sites. The first site of activation is within the tumor itself. After adoptive cell transfer, tumor-specific naive CD8+ T cells have been shown to migrate to the tumor masses, become activated and proliferate there independently of the draining lymph nodes [36]. Mutual activation of DCs and T cells has been shown to occur within tertiary lymphoid structures in tumors, resulting in tumor-specific immunity, which was inhibited by regulatory T cells (Treg) present in the tumor-associated tertiary lymphoid structures. Depleting Treg enhanced anti-tumor response [37]. We believe the second site of activation is in the draining lymph nodes. DCs pick up and process tumor antigens within the tumors and travel to the draining lymph nodes for presentation to naïve and resting T cells. Further, it is possible that some of the Ipi/IL-2 injected IT drains to the locoregional lymph nodes as similarly seen in sentinel lymph node mapping procedure [38], resulting in further activation of T cells in the lymph nodes. An interesting observation was the lack of immune related toxicity, which is probably due to the miniscule amount of Ipi used in our study and possibly due to the inability of Ipi to penetrate the vasculature from the injection site. This suggests that the systemic effect of the treatment was due to trafficking of the activated lymphocytes rather than a leak of some of the Ipi into the systemic circulation.

Another phenomenon observed with cancer immunotherapy is pseudoprogression due to inflammatory changes in the tumor, which is reported in 7.2% of melanoma patients treated with pembrolizumab [39]. In our study, this was observed in two patients (8 and 11). Therefore, physicians and investigators should be aware of the difficulty in accurate assignment of response status during treatment with immunotherapy, whether it is systemically or locally administered.

The relative contributions of IL-2 and Ipi in our treatment model are unclear. A previous preclinical study in mice showed that IT anti-CTLA-4 antibody depleted regulatory T cells and enhanced antitumor immunity and improved survival only when combined with anti-CD25 antibody [40]. Therefore, it is possible that IT injection of Ipi as a single agent may be insufficient to generate systemic immune responses and may require the addition of other agents such as IL-2, anti-PD-1, anti-PD-L1, or anti-OX40.

In our trial in which only one lesion was injected with Ipi/IL-2, we observed complete or partial regression of the injected lesion in 66.7% of our patients. This is comparable to the results seen with IT IL-2 alone [27, 28]. However unlike those studies our observation of strong abscopal effect is quite promising. These responses were correlated with an increase in the frequency of total peripheral IFN-γ or Tbet producing CD+8 T cells in 7 of 10 patients. This result suggests that local intratumoral therapy with IL-2 and Ipi engendered a systemic immune response in these patients.

## MATERIALS AND METHODS

Patients were eligible if they had unresectable stage III, resectable but declined resection or stage IV melanoma with accessible cutaneous, subcutaneous, and/or nodal lesions, according to the AJCC Staging Manual, 7th Edition, 2011. Other inclusion criteria were age of at least 18 years; Eastern Cooperative Oncology Group (ECOG) performance status of 0 to 2; adequate bone marrow, kidney and liver function. Patients agreed to use an appropriate method of birth control while on study. Exclusion criteria were concurrent therapy with any other non-protocol anti-cancer therapy; prior local therapy within 2 weeks or prior systemic therapy within 4 weeks of starting protocol treatment, history of any other malignancy requiring active treatment, pre-existing autoimmunity, clinically significant cardiovascular disease, active systemic infection or known history of HIV infection or chronic hepatitis B or C.

The protocol was approved by the institutional review board at the University of Utah and was conducted in accordance with the ethical principles originating from the Declaration of Helsinki and with Good Clinical Practice as defined by the International Conference on Harmonization. All patients gave written informed consent. The trial was registered withhttp://www.ClinicalTrials.gov identifier NCT01672450.

### Study design

This was a single center, open label phase I dose escalation study. This study assessed the highest tolerable intratumoral dose of Ipi with IL-2. The objective was to primarily assess the safety of the drug combination and to obtain preliminary data on the clinical efficacy of the combination.

The standard 3+3 design was used for the dose escalation phase. Patients were accrued to each dose level in cohorts of up to 3-6 patients. Escalation continued until a dose limiting toxicity (DLT) was observed or the highest dose-level was reached. The goal was to ensure the safety and tolerability and not to determine the maximum tolerated dose (MTD). The study was to test three dose levels of 0.5 mg/0.1 mL (dose level +1), 1 mg/0.2 mL (dose level +2), 2 mg/0.4 mL (dose level +3) Ipi, with a fixed dose of IL-2 (3 mIU).

Patients were treated with Ipi on day 1 of every week for 8 weeks and IL-2 on days 1, 3, 5 on weeks 1-2 and days 1 and 4 of weeks 3-8. Drugs were administered IT, using separate 30-gauge needles for superficial injections. Intra-patient dose escalation was not permitted since toxicity was monitored throughout the duration of treatment and follow-up.

### Immune characterization

Peripheral blood mononuclear cells (PBMCs) were isolated from patient whole blood samples using Ficoll-Paque (GE Healthcare) gradient centrifugation. Isolated PBMCs were resuspended in freezing media (45% DMEM 45% FBS 10% DMSO) and frozen. For cellular restimulation and intracellular cytokine stains, frozen PBMCs were rapidly thawed and washed with RP-10 (RPMI media + 10% FBS + pen/strep) then resuspended in RP-10 and transferred to a 96-well plate. Brefeldin A was added to a concentration of 10 uM (BD GolgiPlug^TM^), as was PMA (20 ng/mL) and ionomycin (1ug/mL) to stimulate cytokine production [41–43]. The analysis was performed as described by manufacturer.

### Assessments

Each adverse event (AE) was evaluated to determine the severity grade based on National Cancer Institute's Common Terminology Criteria for Adverse Events (CTCAE version 4.0, grade 1-5). Relationship of AE was assessed for IL-2 and Ipi and whether it constituted an immune related AE or serious adverse event (SAE). Protocol guidelines for the management of immune-related AE included the administration of corticosteroids (orally or intravenously), a delay in a scheduled dose, or discontinuation of therapy.

Dose-limiting toxicity (DLT) was defined as any of the following AE/SAE as assessed in the reporting period by the investigator: Any grade > 3 (non-autoimmune) toxicity suspected to be related to the study drugs, any grade 3 autoimmune event that did not resolve with intervention (steroids), to a grade 1 or less within 21 days. A grade 3 ulcerated or necrotic lesion located at the injection site was not to be considered a DLT. Patients who were not assessable for toxicity in the first three weeks were to be replaced, however patients not assessable for efficacy were not substituted.

### Statistical analysis

The primary endpoint was to assess the safety data of all patients receiving at least 1 dose of study treatment. The secondary end points were to assess the clinical response of the treated and untreated lesions. Secondary endpoints were summarized for all treated patients together. There was no formal hypothesis tests for secondary endpoints. The disease control rate, overall response rate and response rate was summarized by the observed proportion and exact 95% binomial confidence interval. PMBC was analyzed for T cell subsets using flow cytometry. The change in the fraction of T cells was reported from baseline to the eighth week of treatment.

## CONCLUSIONS

IT Ipi/IL-2 given to patients with non resectable stage III and IV melanoma is well tolerated. Most toxicities were Grade 1 or 2 in nature (fatigue, headache, pain, chills, rash, etc). The clinical benefit rate was 50%, with 10% CR, 30% PR and 10% SD. A PD turned out to be a pseudoprogression with a pathologic CR. An abscopal effect was seen in 8/9 patients (89%). Pseudoprogression was observed in two patients. We plan to conduct a phase II trial using IT Ipi/IL-2 in conjunction with systemic immunotherapy such as an anti-PD-1 or PD-L1 monoclonal antibody.

## SUPPLEMENTARY MATERIALS FIGURES


