# Supplementary material for: A phase I study of intratumoral ipilimumab and interleukin-2 in patients with advanced melanoma
Source: Oncotarget. 2016 Jul 6;7(39):64390–9. doi: 10.18632/oncotarget.10453 (PMC5325451; doi:10.18632/oncotarget.10453)
Supplement: Supplementary file 1 [file oncotarget-07-64390-s001.pdf]

A phase I study of intratumoral ipilimumab and interleukin-2 in patients with advanced melanoma

Supplementary Material

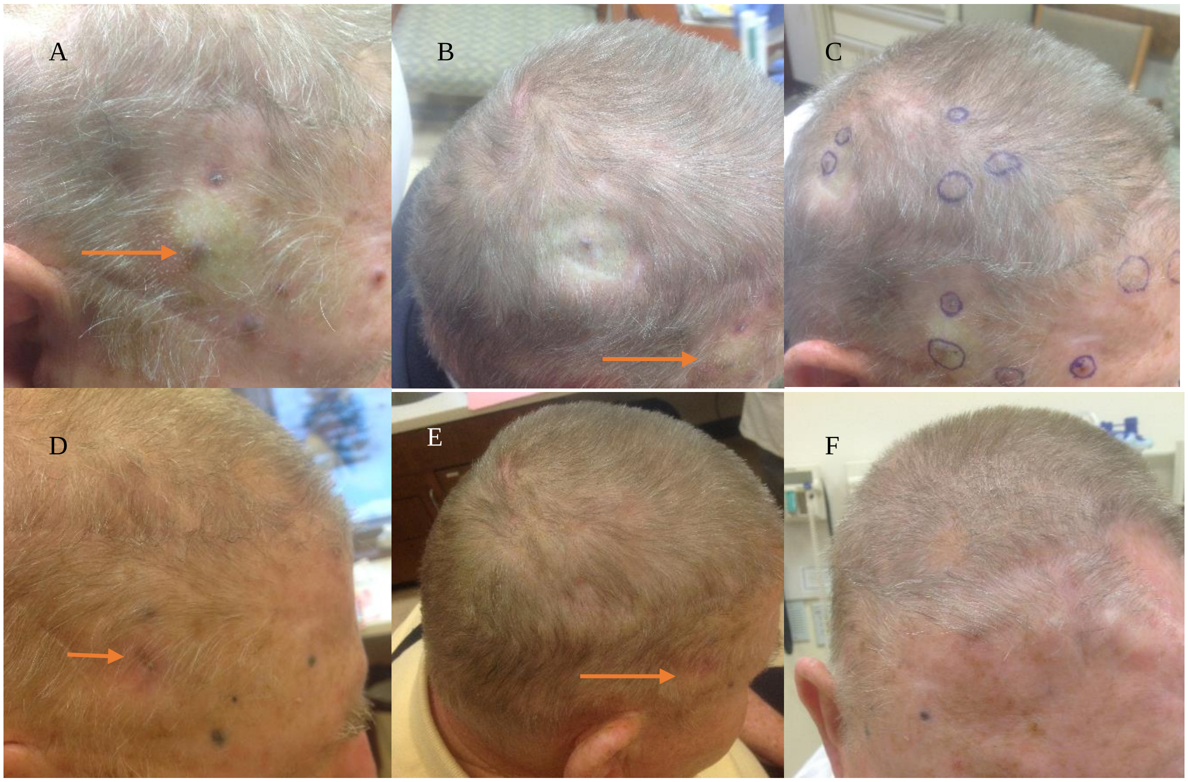

Figure 1: Photographs A, B and C of patient 3 with recurrent melanoma with multiple lesions on the scalp and forehead at baseline prior to treatment with Ipi/IL-2. Photograph A is the site of the injected lesion which was 1.2 x 1.2 cm. There were 4 lesions that were larger than 1 cm and more than 20 cutaneous and subcutaneous lesions in total. The right temporal lesion was injected at the starting dose level +1 with IT Ipi/IL-2. The arrow indicates the injected lesion. D, E and F are corresponding photographs1 month post treatment. Injected lesion and two other large non injected lesions disappeared along with multiple smaller lesions.

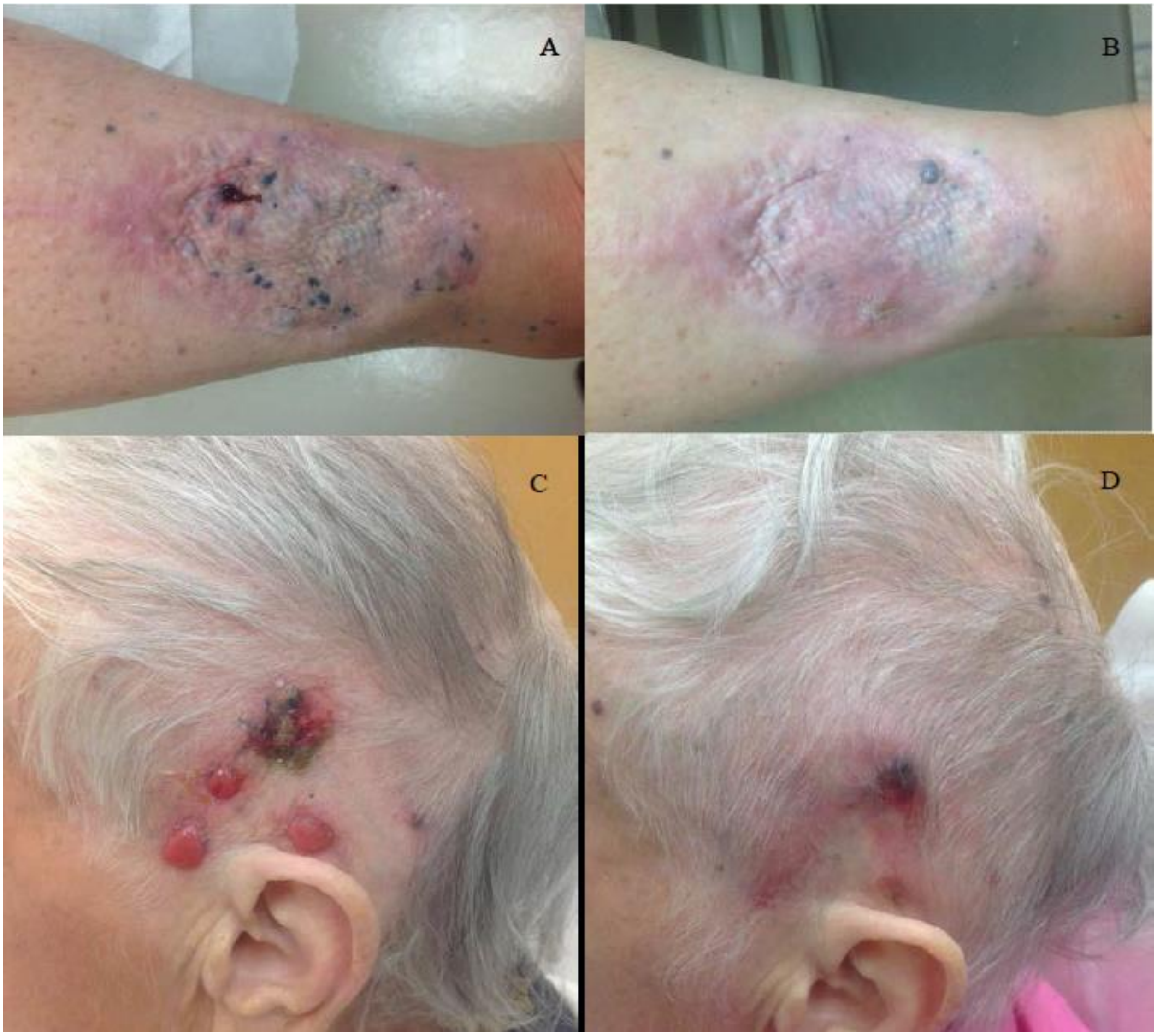

Figure 2: Photographs A and C of patients 4 and 5 with melanoma at baseline prior to treatment with Ipi/IL-2. Both patients were at dose level 2. Photographs B and D are the corresponding patients one month post treatment with intratumoral ipi/IL-2. Both patients were positive to disappearance of the injected lesion and abscopal effect. Patient 4 (A and B) was evaluated for the primary endpoint of toxicity, but was unevaluable for the secondary endpoint of efficacy. Patient 5 (C and D) had PR, but subesquently progressed.
